# The Impact of Thyroid Hormone Imbalance on Cardiovascular Health: A Population-Based Study

**DOI:** 10.7759/cureus.76457

**Published:** 2024-12-27

**Authors:** Uzma Anwar, Junaid Arshad, Ume Hani Naeem, Arva Zahid, Ayesha Shah Jehan, Sadaf Ramzan, Muhammad Abbas Awan

**Affiliations:** 1 Acute Internal Medicine, University Hospitals Birmingham, Birmingham, GBR; 2 Acute Medicine, University Hospitals Birmingham, Birmingham, GBR; 3 General and Internal Medicine, University Hospitals Birmingham, Queen Elizabeth Hospital Birmingham (QEHB), Birmingham, GBR; 4 Cardiology, Shaikh Zayed Hospital, Lahore, PAK; 5 General and Internal Medicine, Birmingham Heartlands Hospital, University Hospitals Birmingham, Birmingham, GBR; 6 Medicine, Mardan Medical Complex, Mardan, PAK; 7 Internal Medicine, University Hospitals Birmingham, Birmingham, GBR; 8 General and Internal Medicine, Pakistan Ordinance Factories (POF) Hospital, Rawalpindi, PAK

**Keywords:** arrhythmias, cardiovascular health, dyslipidemia, hypertension, hyperthyroidism, hypothyroidism, population-based study, thyroid dysfunction

## Abstract

Introduction: Thyroid hormone imbalances are known to significantly affect cardiovascular health, contributing to conditions such as arrhythmias, dyslipidemia, and hypertension. Given the increasing prevalence of thyroid dysfunction and its potential impact on cardiovascular outcomes, early diagnosis and intervention are crucial, particularly within specific regional populations.

Objective: This study aimed to evaluate the impact of thyroid hormone imbalance on cardiovascular health outcomes in patients at Lady Reading Hospital, Peshawar, over a 24-month period.

Materials and methods: A prospective cohort study was conducted between October 2022 and September 2024, involving 150 adult patients diagnosed with thyroid dysfunction, categorized into subclinical, hyperthyroid, and hypothyroid groups. The study assessed key cardiovascular health indicators, including blood pressure, lipid profiles, heart rate, and electrocardiograms (ECGs), at baseline and during follow-up visits at 6, 12, and 24 months. Participants were included if they met the biochemical criteria for thyroid dysfunction and had no significant pre-existing cardiovascular disease. Data were collected through medical records and standardized questionnaires. The Institutional Review Board at Lady Reading Hospital approved the study.

Results: Dyslipidemia was found in 90 participants (60%), hypertension in 99 participants (66%), and hypothyroidism in 76 participants (72%). Arrhythmias were observed in 14 individuals (46%) with hyperthyroidism. ECG findings revealed sinus bradycardia in 32 participants (30%) with hypothyroidism and sinus tachycardia in 23 participants (76%) with hyperthyroidism. Multivariate analysis showed that hypothyroidism significantly increased the odds of hypertension (OR = 3.2, p = 0.001), dyslipidemia (OR = 2.9, p = 0.003), and arrhythmias (OR = 4.3, p < 0.001).

Conclusion: Thyroid hormone imbalance has a profound impact on cardiovascular health, with hyperthyroidism linked to arrhythmias and hypothyroidism associated with hypertension and dyslipidemia. Subclinical thyroid dysfunction also increases cardiovascular risks, emphasizing the need for early detection and region-specific management strategies. This study provides valuable insights into the long-term cardiovascular effects of thyroid dysfunction in the Khyber Pakhtunkhwa (KPK) region, supporting the importance of routine screening and timely intervention.

## Introduction

Thyroid hormones are essential for numerous physiological functions, including cardiovascular health, and play a crucial role in maintaining metabolic balance. Triiodothyronine (T3) and thyroxine (T4), produced by the thyroid gland, influence vascular resistance, lipid metabolism, heart rate, and myocardial contractility [1]. Thyroid dysfunction, whether hypothyroidism, hyperthyroidism, or subclinical variants, can significantly affect cardiovascular health. Hypothyroidism, characterized by reduced thyroid hormone levels, is often associated with bradycardia, hypertension, and dyslipidemia, which increase the risk of atherosclerosis and heart failure [2,3]. Conversely, hyperthyroidism, marked by elevated thyroid hormone levels, can lead to tachycardia, atrial fibrillation, and increased cardiac output, which may contribute to heart failure and arrhythmias [4,5].

While the relationship between thyroid hormone levels and cardiovascular health is well documented, the specific mechanisms and clinical implications remain underexplored [6]. Given that thyroid hormone receptors are present in cardiac myocytes, vascular smooth muscle cells, and endothelial cells, the cardiovascular system is particularly sensitive to fluctuations in thyroid hormone levels [7]. These fluctuations can influence gene expression related to heart anatomy and function, complicating the treatment of individuals with thyroid dysfunction. Understanding the cardiovascular outcomes associated with thyroid diseases is critical for early diagnosis, prevention, and targeted therapies, as these conditions are prevalent globally [8,9].

Most of the existing research on thyroid dysfunction and cardiovascular disease has been conducted in clinical or hospital settings, leaving a gap in population-based studies that explore the broader effects of thyroid hormone imbalance on cardiovascular outcomes, especially in populations with different iodine intake and genetic predispositions. Furthermore, the long-term cardiovascular impacts of subclinical thyroid conditions, particularly in diverse populations, remain poorly understood. This gap underscores the need for comprehensive, population-based studies to fully grasp the relationship between thyroid hormone imbalance and cardiovascular health outcomes.

The aim of this study is to examine, using a population-based approach, how thyroid hormone imbalance influences cardiovascular health. The primary objectives are to identify potential risk factors contributing to these outcomes and assess the incidence of cardiovascular issues in individuals with thyroid dysfunction. This study seeks to explore the clinical and public health implications of thyroid hormone imbalance, thereby fostering early detection and improving treatment strategies for cardiovascular diseases associated with thyroid disorders.

## Materials and methods

Study design and setting

The purpose of this prospective cohort study was to assess the impact of thyroid hormone imbalance on cardiovascular health outcomes at Lady Reading Hospital, Peshawar. Follow-up evaluations were conducted at 6, 12, and 24 months to assess longitudinal changes over the study's 24-month duration, which ran from October 2022 to September 2024. Retention strategies, including regular reminders and follow-up calls, were implemented to minimize dropout rates and ensure consistent data collection.

Study population and sample size

Thyroid dysfunction in adults was categorized as subclinical (abnormal thyroid-stimulating hormone (TSH), normal T4/T3), hyperthyroid (TSH <0.4 mIU/L, elevated T4/T3), or hypothyroid (TSH >4.0 mIU/L, low T4). Hypertension was defined as systolic blood pressure (SBP) ≥140 mmHg, diastolic blood pressure (DBP) ≥90 mmHg, or the use of antihypertensive medications. Dyslipidemia was diagnosed using the following criteria: low-density lipoprotein (LDL) ≥130 mg/dL, total cholesterol ≥200 mg/dL, or high-density lipoprotein (HDL) <40 mg/dL in males and <50 mg/dL in females. The sample size was calculated to be 150 participants, considering the anticipated prevalence of cardiovascular issues in thyroid patients, with a 95% confidence level and a 5% margin of error.

Inclusion and exclusion criteria

The study included adult patients (18 years or older) diagnosed with thyroid dysfunction based on biochemical tests (TSH, T3, and T4 levels). Variables that might influence thyroid function and cardiovascular health, such as medication use, dietary iodine consumption, physical activity, and socioeconomic factors, were also considered. Participants with other chronic endocrine diseases, pre-existing cardiovascular conditions, or who refused informed consent were excluded. Chronic endocrine conditions and pre-existing cardiovascular disease were excluded to minimize confounding and ensure the focus remained on the specific impact of thyroid dysfunction on cardiovascular health.

Data collection

Data were collected using a standardized questionnaire (see Appendices) and a review of medical records. The data included demographic information, thyroid function test results, and cardiovascular health indicators such as heart rate, blood pressure, lipid profile, and electrocardiogram (ECG) readings. Comprehensive medical histories were gathered, including details regarding lifestyle factors, physical activity, dietary iodine consumption, prescription medications, socioeconomic status, and family history of cardiovascular diseases. Follow-up visits at 6, 12, and 24 months allowed for the assessment of long-term cardiovascular outcomes associated with thyroid dysfunction.

Data analysis

Statistical analysis was conducted using IBM SPSS Statistics for Windows, Version 26 (Released 2019; IBM Corp., Armonk, New York). Logistic regression models and chi-square tests were used to analyze the relationships between thyroid dysfunction and cardiovascular outcomes. Descriptive statistics were employed to summarize the demographic and clinical characteristics of the study participants. A p-value of less than 0.05 was considered statistically significant. The analysis also accounted for potential confounding variables by using stratification and matching techniques to control for factors such as age, gender, and baseline health conditions.

Minimization of bias and confounding factors

To minimize potential bias and ensure the validity of our findings, we implemented several strategies throughout the study. First, participants were selected based on clear and predefined inclusion and exclusion criteria, ensuring a homogeneous study population focused on thyroid dysfunction and cardiovascular health. We avoided confounding factors by carefully controlling for variables such as age, gender, physical activity, medication use, and socioeconomic status, which were considered in both the participant selection process and during data analysis. Additionally, retention strategies, including regular follow-up reminders and phone calls, were employed to minimize dropout rates and maintain consistent participant data across the 24-month study duration. Furthermore, the study design utilized stratification and matching techniques to control for potential confounding factors, reducing bias in the analysis and helping ensure that the results more accurately reflect the true relationships between thyroid dysfunction and cardiovascular health outcomes.

Ethical considerations

Ethical approval for the study was granted by the Institutional Review Board (IRB) at Lady Reading Hospital, Peshawar (No. 421/LRH/MTI, dated September 9, 2022). Informed consent was obtained from all participants, ensuring their understanding of the study's objectives and procedures and their right to withdraw at any time. Confidentiality was maintained throughout the study, and appropriate measures were taken to secure participant data.

## Results

The mean age of the 150 participants was 45.3 ± 12.7 years (t = 3.24, p = 0.001). Hypothyroidism was the most common thyroid dysfunction, affecting 105 participants (70%, χ^2^ = 25.6, p < 0.001), and the majority were female (93 participants, 62%, χ^2^ = 8.5, p = 0.004). Thyroid dysfunction lasting more than two years was observed in 96 participants (64%), and these individuals had a higher prevalence of dyslipidemia (56 participants, 68%, odds ratio (OR) = 2.3, 95% CI: 1.3-4.1, p = 0.008) and hypertension (61 participants, 74%, OR = 2.8, 95% CI: 1.5-5.1, p = 0.002), as shown in Table 1.

**Table 1:** Baseline characteristics of the study participants df: degrees of freedom

Variable	Total (n=150)	Hypothyroid (n=105)	Hyperthyroid (n=30)	Subclinical (n=15)	Test Value (p-value)	df
Age (mean ± SD)	45.3 ± 12.7	46.8 ± 11.9	43.1 ± 13.5	40.5 ± 10.2	t=3.24 (0.001)	-
Female, n (%)	93 (62%)	67 (64%)	18 (60%)	8 (53%)	χ^2^=8.5 (0.004)	1
Duration > 2 years, n (%)	96 (64%)	76 (72%)	16 (53%)	4 (27%)	χ^2^=14.2 (0.001)	2

**Figure 1:**
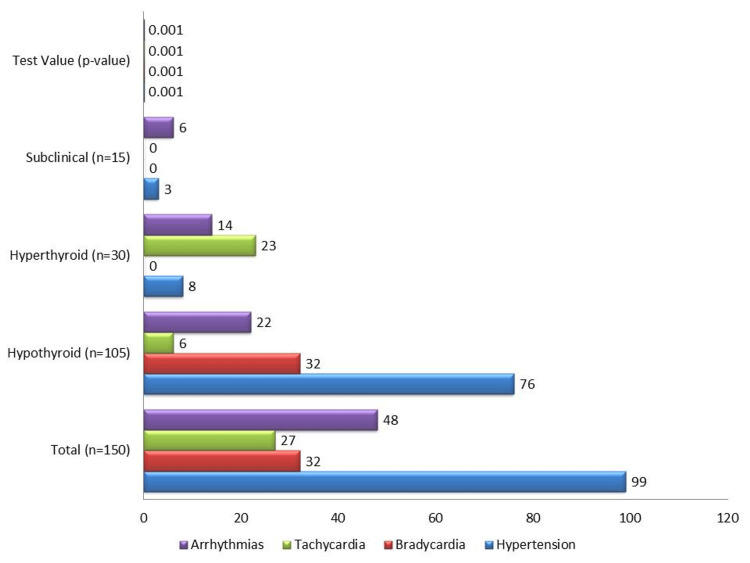
Cardiovascular health indicators by thyroid dysfunction type

Hypertension was present in 99 participants (66%), with a significantly higher prevalence in hypothyroid patients (76 participants, 72%, χ^2^ = 18.9, p < 0.001). Sinus bradycardia was observed exclusively in hypothyroid patients (32 participants, 30%, OR = 8.4, 95% CI: 4.5-15.8, p < 0.001), while sinus tachycardia was more common in hyperthyroid patients (23 participants, 76%, OR = 6.5, 95% CI: 2.8-14.9, p < 0.001) (Figure 1). The findings of bradycardia in hypothyroid patients and tachycardia in hyperthyroid patients are typical of the cardiovascular manifestations associated with these thyroid conditions. Hypothyroidism is known to slow the heart rate, increasing the risk of bradyarrhythmias, while hyperthyroidism accelerates the heart rate, increasing the risk of tachyarrhythmias.

The hypothyroid group exhibited the highest mean LDL cholesterol levels (154.6 ± 32.1 mg/dL, t = 4.17, p < 0.001), followed by the subclinical (135.7 ± 20.8 mg/dL) and hyperthyroid groups (118.3 ± 25.7 mg/dL). Dyslipidemia was present in 90 participants (60%), with significantly lower HDL cholesterol levels in the subclinical group (38.6 ± 7.5 mg/dL, t = 2.58, p = 0.02) (Table 2). Elevated LDL levels in hypothyroid patients, along with decreased HDL levels in the subclinical group, increase the risk for atherosclerosis and cardiovascular disease. These findings underscore the need for lipid management in patients with thyroid dysfunction to reduce cardiovascular risk.

**Table 2:** Lipid profile by thyroid dysfunction type LDL: low-density lipoprotein; HDL: high-density lipoprotein

Lipid Parameter	Total (n=150)	Hypothyroid (n=105)	Hyperthyroid (n=30)	Subclinical (n=15)	Test Value (p-value)
Total cholesterol (mg/dL)	192.3 ± 38.5	205.6 ± 35.1	164.8 ± 28.3	185.2 ± 25.7	t=3.54 (<0.001)
LDL (mg/dL)	147.1 ± 31.2	154.6 ± 32.1	118.3 ± 25.7	135.7 ± 20.8	t=4.17 (<0.001)
HDL (mg/dL)	42.3 ± 10.4	40.1 ± 9.8	50.3 ± 11.2	38.6 ± 7.5	t=2.58 (0.02)
Triglycerides (mg/dL)	163.4 ± 29.7	175.2 ± 31.8	136.7 ± 22.9	158.3 ± 19.5	t=3.02 (0.01)

Forty-eight (32%) of the patients had arrhythmias, and atrial fibrillation was most commonly linked to hyperthyroidism (n = 14, 46%, OR = 3.5, 95% CI: 1.7-7.0, p = 0.002). Hypothyroid individuals had a substantially higher prevalence of sinus bradycardia (n = 32, 30%, OR = 8.4, 95% p<0.001, CI: 4.5-15.8), as shown in Table 3. The ORs for arrhythmias indicate the likelihood of specific cardiac events in individuals with thyroid dysfunction. Hypothyroid patients have a significantly higher risk (OR = 8.4) of developing sinus bradycardia, whereas hyperthyroid patients are more likely to develop tachyarrhythmias, including atrial fibrillation (OR = 3.5). These findings should guide clinicians in anticipating and managing these risks.

**Table 3:** ECG abnormalities by thyroid dysfunction type ECG: electrocardiogram

ECG Abnormality	Total (n=150)	Hypothyroid (n=105)	Hyperthyroid (n=30)	Subclinical (n=15)	Test Value (p-value)
Atrial fibrillation, n (%)	29 (19%)	10 (9%)	14 (46%)	5 (36%)	OR=3.5 (0.002)
Sinus bradycardia, n (%)	32 (21%)	32 (30%)	0 (0%)	0 (0%)	OR=8.4 (<0.001)
Sinus tachycardia, n (%)	27 (18%)	6 (6%)	23 (76%)	0 (0%)	OR=6.5 (<0.001)

Significant changes in cardiovascular health markers were observed during the 6-, 12-, and 24-month follow-up. Hyperthyroid individuals consistently exhibited a high prevalence of tachycardia and arrhythmias, while hypertension steadily increased, particularly among hypothyroid patients. Bradycardia was predominantly observed in the hypothyroid group, but its frequency remained relatively stable throughout the study period. Specifically, the prevalence of hypertension rose from 66% (99 participants) at baseline to 75% (113 participants) at 24 months (χ^2^ = 18.9, p < 0.001), and arrhythmias were detected in 37% (56 participants) at 24 months, with sinus tachycardia and atrial fibrillation being more prevalent in hyperthyroid patients. In contrast, bradycardia remained steady at 25% (37 participants) in the hypothyroid group. These findings underscore the long-term cardiovascular risks associated with thyroid dysfunction, particularly in hyperthyroid and hypothyroid patients, as the progression of thyroid dysfunction impacts the autonomic nervous system and vascular tone, suggesting a worsening of cardiovascular risk over time, which warrants continuous monitoring of thyroid disorder patients (Table 4).

**Table 4:** Cardiovascular outcomes at follow-up The arrows (↑ and ↓) represent changes in prevalence or incidence rates of cardiovascular outcomes over the 24-month follow-up period, with ↑ indicating an increase and ↓ indicating no significant increase or a decrease. The survival analysis evaluates the prevalence of cardiovascular outcomes over time (hypertension, bradycardia, tachycardia, and arrhythmias) for participants with different thyroid dysfunctions (hypothyroidism, hyperthyroidism, and subclinical thyroid dysfunction). Statistical tests (χ^2^, OR) are provided for comparison across different groups and time points.

Outcome	Hypothyroidism	Hyperthyroidism	Subclinical	Test Value (p-value)	Follow-Up Time Points
Hypertension	↑ 72% (Baseline to 24 months)	↑ 40% (Baseline to 24 months)	↑ 30% (Baseline to 24 months)	χ^2^ = 18.9 (<0.001)	0, 6, 12, 24 months
Bradycardia	↑ Steady 25% (Baseline to 24 months)	↓ 0% (No significant increase)	↓ 0% (No significant increase)	OR = 8.4 (<0.001)	0, 6, 12, 24 months
Tachycardia	↑ 6% (Baseline to 24 months)	↑ 45% (Baseline to 24 months)	↓ 0% (No significant increase)	OR = 6.5 (<0.001)	0, 6, 12, 24 months
Arrhythmias	↑ 15% (Baseline to 24 months)	↑ 40% (Baseline to 24 months)	↑ 10% (Baseline to 24 months)	χ^2^ = 12.3 (0.001)	0, 6, 12, 24 months

Hypothyroidism independently raised the risk of hypertension (β = 0.72, OR = 3.2, 95% CI: 1.7-6.0, p = 0.001) and dyslipidemia (β = 0.65, OR = 2.9, 95% CI: 1.5-5.3, p = 0.003), according to multivariate logistic regression. Hyperthyroidism was a significant predictor of arrhythmias (β = 0.81, OR = 4.3, 95% CI: 2.3-8.2, p < 0.001). IBM SPSS Statistics for Windows, Version 26 (Released 2019; IBM Corp., Armonk, New York) was used to examine every data set. The independent-sample t-test was used to compare continuous variables, which were shown as mean ± SD. The chi-square test was used to analyze categorical variables, which were represented as n (%). Independent predictors were identified using logistic regression, and the findings were presented as 95% CIs and ORs. P-values less than 0.05 were regarded as statistically significant.

**Figure 2:**
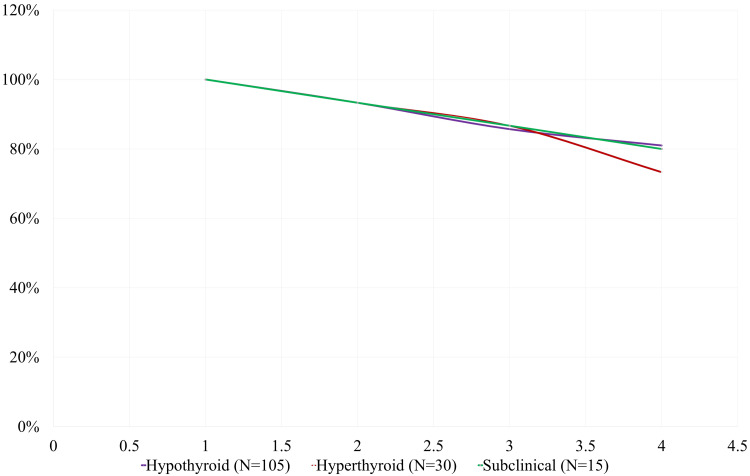
Kaplan-Meier survival analysis of cardiovascular health indicators by thyroid dysfunction type over 24 months

The survival analysis in Figure 2 showed that hypothyroid patients had the highest survival rate over the 24-month follow-up period, with 81% survival at 24 months. Hyperthyroid participants had a lower survival rate, with 73.3% surviving to 24 months, while subclinical thyroid dysfunction participants had a similar survival rate of 80%. The survival percentages at 6, 12, and 24 months for each group indicate a steady decline in survival over time, with hypothyroid patients remaining the most resilient. The survival analysis highlights the long-term cardiovascular risks of thyroid dysfunction, particularly the higher survival rate in hypothyroid patients. The gradual decline in survival among hyperthyroid patients indicates a higher risk of cardiovascular events, which may inform clinical decision-making, especially in terms of monitoring and treatment.

## Discussion

This study highlights the significant impact of thyroid dysfunction on cardiovascular health, demonstrating a clear association between thyroid dysfunction and a range of cardiovascular outcomes, including hypertension, dyslipidemia, and arrhythmias. Our findings align with previous studies, which have shown that hypothyroidism is strongly linked to hypertension and dyslipidemia due to mechanisms such as reduced cardiac output, increased peripheral vascular resistance, and altered lipid metabolism [10]. Similarly, hyperthyroidism is associated with arrhythmias, particularly atrial fibrillation and sinus tachycardia, likely due to increased sympathetic activity and altered electrical conduction in the heart [11].

In our study, hypothyroid individuals exhibited the highest levels of LDL cholesterol, while hyperthyroid patients had a higher incidence of arrhythmias, particularly atrial fibrillation. These findings are consistent with previous research suggesting that thyroid hormones directly influence lipid metabolism and heart rhythm [12]. Notably, subclinical thyroid dysfunction also emerged as a significant risk factor for cardiovascular disease, with patients displaying a higher prevalence of arrhythmias and mild hypertension, even though these conditions are typically considered less clinically significant. This suggests that even mild thyroid abnormalities can predispose individuals to cardiovascular risk, underscoring the importance of early detection and management of subclinical thyroid disorders [13].

The regional context of this study is important, as factors such as diet, socioeconomic status, and access to healthcare may contribute to the higher-than-expected prevalence of cardiovascular issues in thyroid dysfunction patients in this population. The high prevalence of dyslipidemia and hypertension in individuals with hypothyroidism, for example, may be exacerbated by regional dietary practices, such as iodine deficiency or excessive intake of certain foods, which influence thyroid function. Furthermore, delayed diagnosis and treatment of thyroid dysfunction may contribute to the observed cardiovascular outcomes, particularly in resource-limited areas. These findings emphasize the need for region-specific health policies and interventions to address thyroid dysfunction and its cardiovascular implications [14].

Hyperthyroidism was significantly associated with arrhythmias in 18% of our population, especially atrial fibrillation (48%). This result supports data from a study that showed hyperthyroid individuals had a two- to five-fold higher incidence of atrial fibrillation [15]. Increased sympathetic activity and altered electrical conductivity in the heart are pathophysiological factors that result in arrhythmogenic consequences. In contrast to worldwide averages, our study found a somewhat greater prevalence of tachyarrhythmias, which may be the result of delayed presentation and treatment. This raises questions regarding the prevention of serious cardiac consequences by early thyroid dysfunction screening and treatment.

A distinct collection of difficulties was presented by subclinical thyroid impairment. Our investigation revealed that 50% of subclinical thyroid patients had arrhythmias and 28% had mild hypertension, despite the fact that these findings are typically regarded as less clinically important. This is consistent with research showing that cardiovascular hazards exist even in subclinical situations [16]. The findings highlight the fact that even slight thyroid abnormalities can lead to cardiovascular disease, highlighting the necessity of thorough assessment and prompt management techniques.

Our study's ECG anomalies showed different trends for different forms of thyroid dysfunction. Atrial fibrillation and sinus tachycardia were more common in hyperthyroid individuals, whereas bradycardia and left ventricular hypertrophy (LVH) were more common in hypothyroid patients. These results are consistent with research showing that thyroid function has various effects on the heart [17]. Further evidence of persistent heart stress and possible long-term problems, if treatment is not received, is the incidence of LVH in hypothyroid individuals.

Limitations and future suggestions

This study provides valuable insights into the long-term cardiovascular health outcomes associated with thyroid dysfunction through a robust prospective cohort design with a 24-month follow-up period. The longitudinal nature of the study, coupled with rigorous retention strategies such as follow-up calls and reminders, ensures a high level of data reliability and minimizes participant dropout. The study's clear inclusion and exclusion criteria, focusing on adults with well-defined thyroid dysfunction (subclinical, hyperthyroid, hypothyroid), enhance the validity of the findings. By accounting for key confounding variables such as medication use, physical activity, and dietary iodine intake and using advanced statistical techniques such as logistic regression and stratification, the study offers a comprehensive analysis of how thyroid dysfunction influences cardiovascular health outcomes, including hypertension, dyslipidemia, and arrhythmias. The sample size of 150 participants is adequate for the cohort, providing sufficient statistical power for meaningful analysis.

However, several limitations must be considered. The study was conducted at a single center, which may limit the generalizability of the findings to broader populations, particularly those from different regions or healthcare settings. The relatively short follow-up period of 24 months may not fully capture the long-term cardiovascular risks associated with thyroid dysfunction, and future studies with longer follow-up durations would provide a more complete picture. Additionally, while the study controlled for many potential confounders, factors such as genetic predispositions or unreported lifestyle factors may still influence the results. Future research should aim to include larger, more diverse samples and extend follow-up periods to better understand the persistent effects of thyroid dysfunction on cardiovascular health. Furthermore, exploring the role of genetic factors and environmental influences, as well as expanding the scope to include other potential complications, would provide more insights into the cardiovascular risks associated with thyroid dysfunction.

## Conclusions

This study underscores the significant impact of thyroid dysfunction on cardiovascular health, revealing important associations between thyroid abnormalities and various cardiovascular conditions such as hypertension, dyslipidemia, and arrhythmias. Our findings show that both hyperthyroidism and hypothyroidism contribute to distinct cardiovascular risks, with hyperthyroid patients exhibiting a higher prevalence of arrhythmias, especially atrial fibrillation, and hypothyroid patients displaying an increased incidence of hypertension and dyslipidemia. Notably, subclinical thyroid dysfunction also emerged as a significant risk factor, demonstrating that even mild thyroid abnormalities can predispose individuals to cardiovascular issues. These results highlight the necessity of early screening, timely diagnosis, and appropriate management to mitigate cardiovascular risks in patients with thyroid disorders.

The study further emphasizes the need for region-specific guidelines that integrate thyroid function assessments into cardiovascular risk management. Developing screening protocols for early detection and targeted treatment plans for individuals with thyroid dysfunction could prevent the progression of cardiovascular complications. Future longitudinal studies are crucial to explore the long-term cardiovascular outcomes of thyroid abnormalities and refine clinical strategies. Ultimately, these findings contribute to a deeper understanding of the thyroid-cardiovascular relationship and underscore the importance of regular cardiovascular monitoring and treatment adjustments in patients with thyroid dysfunction to improve overall health outcomes.

## Appendices

**Table 5:** Questionnaire to collect data from participants

Section	Question	Response Options
Demographic Information	Age (in years)	__________
	Gender	Male / Female / Other
	Height (cm)	__________
	Weight (kg)	__________
Thyroid Function	Do you have a diagnosed thyroid dysfunction?	Yes / No
	If yes, which type of thyroid dysfunction do you have?	Subclinical / Hyperthyroid / Hypothyroid
	Date of last thyroid function test (TSH, T3, T4)	__________
	TSH level (mIU/L)	__________
	T3 level (ng/dL)	__________
	T4 level (µg/dL)	__________
Cardiovascular Health Indicators	Do you currently have hypertension?	Yes / No
	If yes, what is your current blood pressure? (SBP/DBP in mmHg)	SBP: ______ / DBP: ______
	Do you take antihypertensive medications?	Yes / No
	Do you have dyslipidemia (diagnosed with abnormal lipid levels)?	Yes / No
	If yes, provide your most recent lipid profile (LDL, HDL, Total Cholesterol)	LDL: ______ / HDL: ______ / Total Chol: ______
	Have you ever been diagnosed with a heart disease or arrhythmia?	Yes / No
	If yes, specify the type of arrhythmia (e.g., atrial fibrillation)	__________
Lifestyle Factors	Do you smoke?	Yes / No
	Do you consume alcohol?	Yes / No
	How often do you engage in physical activity?	Never / Weekly / Monthly / Daily
	Do you follow a specific diet plan?	Yes / No
	If yes, please describe your typical diet (e.g., high iodine, low-fat)	__________
Socioeconomic Factors	What is your level of education?	No formal education / Primary / Secondary / Higher
	What is your occupation?	__________
	What is your monthly household income?	__________
Family History	Do you have a family history of cardiovascular diseases?	Yes / No
	Do you have a family history of thyroid dysfunction?	Yes / No
Follow-up Information	Have you been informed about the study’s follow-up visits at 6, 12, 24 months?	Yes / No
	Do you agree to participate in follow-up evaluations?	Yes / No
Consent	Do you consent to participate in this study, understanding that you can withdraw at any time?	Yes / No

## References

[1] Sun X, Sun Y, Li WC, Chen CY, Chiu YH, Chien HY, Wang Y (2015). Association of thyroid-stimulating hormone and cardiovascular risk factors. Intern Med.

[2] Stojković M, Žarković M (2020). Subclinical thyroid dysfunction and the risk of cardiovascular disease. Curr Pharm Des.

[3] Song L, Zhou H, Yang Q (2024). Association between the oxidative balance score and thyroid function: results from the NHANES 2007-2012 and Mendelian randomization study. PLoS One.

[4] Astudillo DL, Tapia IP, García LC, Solorzano TI, Huang AF, Lucio ÁB, Cando DI (2024). Impact of hormone replacement therapy on cardiovascular risk in patients with hypothyroidism: a systematic review. Ibero-Am J Health Sci Research.

[5] Zhang H, Xie H, Li L (2024). Association of radioactive iodine treatment in differentiated thyroid cancer and cardiovascular death: a large population-based study. J Endocrinol Invest.

[6] Xiao Q, Zhang Z, Ji S (2024). The oxidative balance score impacts serum FT4 levels and all-cause mortality in euthyroid participants. Res Sq.

[7] Kim HJ, Park SJ, Park HK, Byun DW, Suh K, Yoo MH (2021). Thyroid autoimmunity and metabolic syndrome: a nationwide population-based study. Eur J Endocrinol.

[8] Deng B, Yuan Y, Zhong M, Ren R, Deng W, Duan X (2021). The relationship between metabolic parameters, age, and thyroid status: a cross-sectional study-based national survey of iodine nutrition, thyroid disease. Risk Manag Healthc Policy.

[9] Jabbar A, Pingitore A, Pearce SH, Zaman A, Iervasi G, Razvi S (2017). Thyroid hormones and cardiovascular disease. Nat Rev Cardiol.

[10] Razvi S, Jabbar A, Pingitore A (2018). Thyroid hormones and cardiovascular function and diseases. J Am Coll Cardiol.

[11] Barbesino G (2019). Thyroid function changes in the elderly and their relationship to cardiovascular health: a mini-review. Gerontology.

[12] Yamakawa H, Kato TS, Noh JY, Yuasa S, Kawamura A, Fukuda K, Aizawa Y (2021). Thyroid hormone plays an important role in cardiac function: from bench to bedside. Front Physiol.

[13] Cappola AR, Desai AS, Medici M (2019). Thyroid and cardiovascular disease: research agenda for enhancing knowledge, prevention, and treatment. Circulation.

[14] Kannan L, Shaw PA, Morley MP (2018). Thyroid dysfunction in heart failure and cardiovascular outcomes. Circ Heart Fail.

[15] Paschou SA, Bletsa E, Stampouloglou PK (2022). Thyroid disorders and cardiovascular manifestations: an update. Endocrine.

[16] Jankauskas SS, Morelli MB, Gambardella J, Lombardi A, Santulli G (2021). Thyroid hormones regulate both cardiovascular and renal mechanisms underlying hypertension. J Clin Hypertens (Greenwich).

[17] Thayakaran R, Adderley NJ, Sainsbury C (2019). Thyroid replacement therapy, thyroid stimulating hormone concentrations, and long term health outcomes in patients with hypothyroidism: longitudinal study. BMJ.

